# Conventional and living guideline for schizophrenia: barriers and facilitating factors in guideline implementation

**DOI:** 10.1007/s00406-023-01663-1

**Published:** 2023-08-15

**Authors:** Carolin Lorenz, Duygu Güler, Theresa Halms, Naiiri Khorikian-Ghazari, Astrid Röh, Marisa Flick, Angelika Burschinski, Charline Pielenz, Eva Salveridou-Hof, Thomas Schneider-Axmann, Marco Schneider, Elias Wagner, Peter Falkai, Wolfgang Gaebel, Stefan Leucht, Alkomiet Hasan, Gabriele Gaigl

**Affiliations:** 1 Department of Psychiatry, Psychotherapy and Psychosomatic, Medical Faculty, University of Augsburg, BKH Augsburg, Augsburg, Germany; 2 Department of Psychiatry and Psychotherapy, Klinikum Rechts Der Isar, School of Medicine, Medical Faculty, Technical University of Munich, 81675 Munich, Germany; 3 Department of Psychiatry and Psychotherapy, Medical Faculty, LVR-Hospital Düsseldorf, Heinrich-Heine-University, Düsseldorf, Germany; 4 WHO Collaborating Centre on Quality Assurance and Empowerment in Mental Health, DEU-131 Düsseldorf, Germany; 5 Department of Psychiatry and Psychotherapy, University Hospital, LMU Munich, Munich, Germany

**Keywords:** Living guideline, Guideline implementation, Schizophrenia, MAGICapp, Barriers

## Abstract

This study aims to investigate the barriers and facilitators to guideline adherence for the print format of the German schizophrenia guideline as well as for the concept of a digital living guideline for the first time. For this purpose, the schizophrenia guideline was transferred to a digital guideline format within the web-based tool MAGICapp. An online survey was performed under participation of mental healthcare professionals (medical doctors, psychologists/psychotherapists, psychosocial therapists, caregivers) in 17 hospitals for psychiatry in Southern Germany and a professional association for German neurologists and psychiatrists. 524 participants opened the survey, 439 completed the demographic questions and commenced the content-related survey and 309 provided complete data sets. Results indicate a higher occurrence of knowledge-related barriers for the living guideline. The print version is associated with more attitude-related and external barriers. Older professionals reported more attitude-related barriers to a living guideline compared to younger professionals. Differences between professions regarding barriers were found for both formats. Various barriers exist for both guideline formats and a need for facilitators was expressed across professions. Many of the mentioned obstacles and facilitators can be more easily addressed with living guidelines. However, also living guidelines face barriers. Thus, the introduction of these new formats alone cannot lead to sustainable behavior change regarding guideline adherence. Yet, living guidelines seem to be a cornerstone to improved and tailored guideline implementation as they facilitate to keep recommendations up to date and to address the need of individual professional groups.

**Supplementary Information:**

The online version contains supplementary material available at 10.1007/s00406-023-01663-1.

## Introduction

Schizophrenia is a severe and often life-long disorder which ranks among the 20 leading causes of disability and grades 20th in terms years lived with disability (YLDs) overall according to the recent Global Burden of Disease report [1].

Due to the high burden of the disease for patients living with schizophrenia and relatives as well as the high economic costs, evidence-based guidelines are crucial for ensuring that patients receive the treatment as needed. However, implementation of treatment guidelines into clinical practice faces many difficulties and is insufficient worldwide [2–5] as well as in Germany, where a recent study [6] indicated an unsatisfactory implementation of the German evidence- and consensus-based guideline for schizophrenia published in 2019 [7, 8]. Consequently, the question arises how implementation of guidelines can be improved and thereby reduce the evidence-practice-gap. First, behavioral changes among healthcare professionals are required [5, 9]. The sequence of behavior change ideally preceding guideline adherence is described by Cabana’s Knowledge-Attitude-Behavior Framework, according to which physicians’ knowledge is affected initially, then attitudes and finally behavior [9]. Each of these categories is assigned with various barriers impeding guideline adherence and underlines the importance of identifying obstacles and possible facilitating factors. Thereby reasons why physicians do not adhere to clinical guidelines can be revealed and targeted solution approaches to improve guideline adherence may be developed. Barriers regarding physicians’ knowledge are e.g., lack of awareness or lack of experience, while obstacles with respect to physicians’ attitude are related to e.g., lack of motivation or deficient benefits for everyday clinical work. Clinicians’ behavior is influenced by patient-, guideline-, or environmental-related factors, such as rejection of the guideline by patients or lack of time resources. Second, so-called living guidelines could address the problem of rapidly increasing medical knowledge, which means that guidelines are often out of date by the time they are published [10–13]. Thus, guideline adherence is hampered due to the situation that recommendations do not correspond to the current state of the art. In contrast, with living guidelines individual recommendations can be updated as soon as relevant new evidence is available [10]. In that regard, the user’s perception on the concept of living guidelines has not yet been explored.

The current German guideline for schizophrenia is supposed to gradually be converted into a living guideline [14]. Therefore, the guideline was integrated into the web-based evidence ecosystem MAGICapp facilitating the entire process of creating a living guideline [15].

This study aims to elaborate for the first time the anticipated barriers and facilitating factors to guideline adherence for both the classical print version of the German guideline for schizophrenia and an upcoming living guideline. Moreover, preferences of healthcare professionals in the use of living guidelines will be presented.

## Design and methods

### Subjects and recruitment

**Fig. 1:**
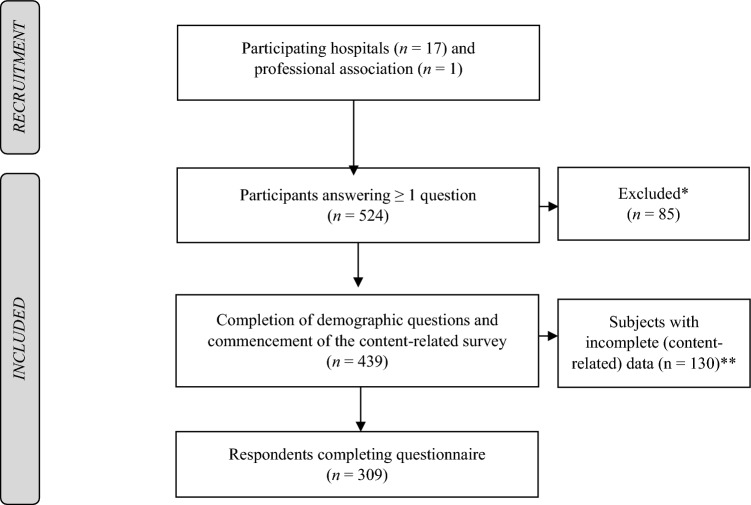
Recruitment and study flowchart. *Participants were excluded due to missing experience on the treatment of mental disorders (*n* = 22) or the absence of answering at least one content-related question (*n* = 63). ** Participants were counted as “drop-outs” if not completing the content-related survey. Data were used for analysis until the participant’s drop-out

A cross-sectional online survey was conducted from January 2022 to April 2022 in the context of a larger project (Structured implementation of digital, systematically updated guideline recommendations for enhanced therapeutic adherence in schizophrenia, SISYPHOS project) [16]. The focus of our preceding paper on this topic was the implementation status of the guideline for schizophrenia and the attitude toward an upcoming living guideline [17]. There are no duplications of results between the two papers. In total, 17 hospitals for psychiatry, psychotherapy and psychosomatic medicine in Southern Germany (see Supplementary Table 1) and one professional association for German neurologists and psychiatrists (BVDN: Berufsverband Deutscher Nervenärzte e. V.) [18] took part in the study by forwarding the link to their clinical staff (medical doctors, psychologists/psychotherapists, psychosocial therapists, caregivers (e.g., nurses)) and members. We used the licensed LimeSurveyR version 5.3.4 + (LMU hospital) to create the questionnaire, perform the survey, and ensure an anonymous participation. A reminder mail was sent to the participating hospitals after approximately three weeks. The data protection officer of the University Hospital Munich reviewed the survey, and the local ethical committee approved the project (reference number 21–0780). The trial has been performed according to the latest version of the Declaration of Helsinki [19]. If not defined otherwise, the term “schizophrenia guideline” refers to the current German evidence- and consensus-based guideline for schizophrenia 2019 [7]. Figure 1 shows the recruitment and study flowchart.

### Survey structure

The survey aimed to evaluate the implementation of the general guideline for schizophrenia as well as of four key recommendations [17]. Moreover, the survey was designed to investigate the attitude toward an upcoming living guideline for schizophrenia and explore perceived barriers (questions 42–55) and facilitators (questions 56–70) regarding knowledge, attitude and behavior [9] of the implementation of the schizophrenia guideline. For our analysis, we allocated these questions to the three sequences of behavior change preceding guideline adherence according to Cabana’s Knowledge–Attitude–Behavior framework [9]. Examples from the questionnaire examining knowledge-related barriers are e.g., “I have heard of the corresponding guideline format before”, for knowledge-related facilitators e.g., “I would like to have (more) training/education on working with the guideline format”. Attitude-related barriers were illustrated with e.g., “I lack motivation to deal with the guideline format”, attitude-related facilitators with e.g., “I would like to have clinical conditions more considered (e.g., comorbidities, complex courses) in the content of the guideline”. With statements such as “Due to lack of time resources (e.g. due to a high workload) the use of the guideline format seems to be difficult”, behavior-related barriers were investigated, whereas behavior-related facilitators were represented with e.g., “I would like to have short, clear treatment checklists”).

Barriers and facilitators were examined on a five-point Likert scale (agreement: 1 = strongly disagree, 2 = disagree, 3 = neutral, 4 = agree, 5 = strongly agree) for both formats: print and living. As no living guideline for mental disorders was available at the time of the study [20], the concept of a living guideline was introduced to the participants (1) by an explanatory text and (2) visualized by screenshots of the unpublished living guideline for schizophrenia. The presented text, screenshots and the whole questionnaire are displayed in the supplement. Moreover, the preferences of healthcare providers when using living guidelines were investigated (questions 71–79). The questionnaire was provided in German language and translated into English by the authors for this publication.

### Statistical analysis

All analyses were carried out in IBM SPSS for Windows (version 29) with a significance level of α = 0.05. Descriptive statistics are displayed with frequency and percentage distributions for binary data. For continuous data, means and standard deviations are presented and additionally medians for categorical data. Intergroup differences were assessed using Chi^2^ tests in case of binary data. Kruskal–Wallis tests for between group analyses (Dunn–Bonferroni tests for subgroup analyses to account for multiple testing in case of significant intergroup differences) or Wilcoxon signed-rank tests (in case of dependent samples within subjects) were used for categorical data (e.g., Likert scale). In addition to age groups [young (20–34 years old) vs. middle-aged (35–49 years old) vs. older mental healthcare professionals (50–66 years old)], professional groups were compared (medical doctors vs. psychotherapists/psychologists vs. psychosocial therapists vs. caregivers (e.g., nurses)). See Table 1 for a detailed listing of the associated occupational profiles.

**Table 1:** Descriptive characteristic of participants

	Total *N* = 439	
	*n*	%
**Gender**		
Female	299	68.1%
Male	140	31.9%
Divers	0	0.0%
**Profession**		
*Psychologist / psychotherapist*		
Total	80	18.2%
Psychological psychotherapist	24	5.5%
Psychotherapy trainee	40	9.1%
Psychologist	16	3.6%
*Medical doctor*		
Total	187	42.6%
Specialist for psychiatry and psychotherapy	94	21.4%
Assistant doctor for psychiatry and psychotherapy	78	17.8%
Specialist for psychosomatic medicine and psychotherapy	2	0.5%
Assistant doctor for psychosomatic medicine and psychotherapy	2	0.5%
Specialist for neurology	6	1.4%
Assistant docotor for neurology	3	0.7%
Specialist for general medicine with additional qualification for psychosomatic care	1	0.2%
Assistant doctor for general medicine with additional qualification for psychosomatic care	0	0.0%
Specialist/assistant doctor of other medical fields	1	0.2%
*Psychosocial therapist*		
Total	67	15.3%
Occupational therapist	27	6.2%
Sporttherapist	10	2.3%
Social pedagogue	17	3.9%
Social worker	1	0.2%
Art therapist	12	2.7%
Peer-/Recovery attendant	0	0.0%
Sociotherapist	0	0.0%
*Caregiver*		
Total	96	21.9%
Specialist nurse for psychiatric care	28	6.4%
Qualified nurse	64	14.6%
Remedial nurse (Heilerziehungspfleger:in)	4	0.9%
Other profession	9	2.1%
**Workplace/setting**^*a*^		
*Inpatient setting*		
University hospital	69	15.7%
Public hospital	320	72.9%
Non-profit hospital	28	6.4%
Private hospital	14	3.2%
*Outpatient setting*		
Practice with health insurance license	7	1.6%
Private practice	4	0.9%
Practice within the framework of psychotherapy training	10	2.3%
*Research*	10	2.3%
*Other*	4	0.9%
		M (SD)
**Age**		
Years	439	41.41 (11.62)
	Mdn	M (SD)
**Experience**^*b*^		
Mental disorders	4.00	3.88 (0.91)
Schizophrenic disorders	3.00	3.43 (0.95)

*Note.* ^a^Multiple answers were possible. ^b^Participants were asked how they would rate their experience in treating people with mental disorders or schizophrenic disorders (1 = not at all experienced – 5 = very experienced). *N*  number of participants. *M*  means. *SD*  standard deviations. *Mdn*  medians

## Results

### Participants’ characteristics

524 Participants originally took part in the study meaning they responded to at least one question. 85 respondents were excluded from analyses due to missing experience in the treatment of mental disorders (*n* = 22) or not answering at least one content-related question (*n* = 63). Only participants who completed the demographic questions and answered at least one content-related item were included in the analysis, *N* = 439 (see Fig. 1). Participants were counted as “drop-outs” if not completing the content-related survey (*n* = 130). However, all available data until the participant’s drop-out was used for the analysis.

Table 1 depicts demographic information of the participants. In the Supplementary Tables 2, 3, 4, further demographic information can be found regarding comparisons between included and excluded participants as well as between professions and age groups.

### Barriers to guideline implementation

The investigated barriers for both the print format and the living guideline for schizophrenia were categorized according to the three sequences of behavioral change by Cabana et al. [9]: Knowledge, attitude and behavior.

More than two-third considered “lack of experience” (80%) and “lack of awareness” (64%) for living guidelines in general as barriers in the use of the upcoming living guideline for schizophrenia. Moreover, 64% of respondents reported to anticipate difficulties in accessing the living guideline for schizophrenia once published. Regarding the utilization of the schizophrenia guideline as print version, the most important barrier appeared to be “lack of time resources” (63%) followed by “lack of trainings” (53%). See Table 2 for an overview of the presented barriers and related descriptive information.

**Table 2:** Barriers in guideline utilization for the print and living format of the guideline for schizophrenia

		% Yes	Mdn	M	SD	Wilcoxon test	
						Z	*p*
**Knowledge-related barriers**							
Lack of awareness^a^ [Q42] (*n* = 326)	*Print*	11.7%	2.00	1.96	1.15	12.66	< 0.001
	*Living*	63.5%	4.00	3.67	1.20		
Lack of experience^a^ [Q43] (*n* = 326)	*Print*	23.9%	2.00	2.49	1.25	12.91	< 0.001
	*Living*	80.4%	4.00	4.23	0.82		
Lack of competence [Q45] (*n* = 324)	*Print*	3.1%	2.00	1.85	0.81	3.36	< 0.001
	*Living*	5.9%	2.00	1.96	0.88		
Lack of knowledge about access [Q52] (*n* = 320)	*Print*	19.7%	2.00	2.28	1.12	11.15	< 0.001
	*Living*	64.1%	4.00	3.64	1.03		
Lack of trainings [Q46] (*n* = 324)	*Print*	52.5%	4.00	3.43	1.05	2.28	0.023
	*Living*	54.0%	4.00	3.52	1.07		
Mean—knowledge-related barriers^b^(*n* = 320)	*Print*	22.2%	2.20	2.40	0.76	– 13.76	< 0.001
**Attitude-related barriers**							
	*Living*	53.6%	3.40	3.41	0.53		
Lack of motivation [Q48] (*n* = 323)	*Print*	27.2%	2.00	2.67	1.07	– 5.56	< 0.001
	*Living*	14.9%	2.00	2.40	0.97		
Lack of confidence [Q44] (*n* = 324)	*Print*	3.4%	2.00	1.89	0.80	– 1.05	0.296
	*Living*	2.8%	2.00	1.82	0.77		
Lack of benefits for clinical work [Q47] (*n* = 323)	*Print*	6.5%	2.00	2.25	0.81	– 5.12	< 0.001
	*Living*	4.0%	2.00	2.06	0.77		
Limitation of therapeutical freedom [Q49] (*n* = 323)	*Print*	4.6%	2.00	2.02	0.82	– 2.32	0.020
	*Living*	3.7%	2.00	1.98	0.80		
Mean—attitude-related barriers^b^ (*n* = 323)	*Print*	10.4%	2.25	2.20	0.57	– 6.09	< 0.001
	*Living*	6.4%	2.00	2.06	0.59		
**External barriers**							
Confusing layout/structure [Q50] (*n* = 320)	*Print*	34.1%	3.00	3.13	0.83	– 3.64	< 0.001
	*Living*	31.9%	3.00	2.89	0.98		
Too long/complex [Q51] (*n* = 320)	*Print*	48.4%	3.00	3.44	0.90	– 10.45	< 0.001
	*Living*	7.8%	3.00	2.71	0.68		
Lack of clinical testing [Q53] (*n* = 320)	*Print*	20.9%	3.00	2.66	0.97	6.05	< 0.001
	*Living*	24.1%	3.00	3.02	0.84		
Lack of time resources [Q54] (*n* = 320)	*Print*	62.8%	4.00	3.58	0.94	– 7.26	< 0.001
	*Living*	39.1%	3.00	3.23	0.90		
Rejection by patients [Q55] (*n* = 320)	*Print*	8.8%	3.00	2.51	0.84	0.42	0.674
	*Living*	5.6%	3.00	2.52	0.79		
Mean – External barriers^b^ (*n* = 320)	*Print*	35.0%	3.00	3.07	0.47	– 6.45	< 0.001
	*Living*	21.7%	2.80	2.88	0.46		

*Note. Print* = guideline for schizophrenia in the print-/pdf-format; *Living* = living guideline for schizophrenia*.* Agreement on barriers was assessed by a 5-point Likert scale (1 = *strongly disagree* to 5 = *strongly agree*). *% Yes* represents the percentage of participants, who *agreed* or *strongly agreed* to the statement. *N* = number of participants, *M* = means, *SD* = standard deviations, *Mdn* = medians, *Z* = standard score. As no German living guideline for mental disorders existed at the time of the study, participants were asked to answer the questions based on the visual and written description / presentation of the concept of living guidelines. Numbers of questions are displayed in square brackets. The complete questionnaire is shown in the supplement. ^a^The items referred to any living guideline (e.g., living guideline for somatic disorder). ^b^The variables represent the mean agreement rate on items of the corresponding categories of the “Knowledge-Attitude-Behavior Framework”

*Group comparisons—print versus living:* Wilcoxon tests for dependent variables indicate an increased occurrence of knowledge-related implementation barriers in the context of the living guideline and of external and attitude-related barriers in the implementation of the print format. Higher agreement scores on knowledge-related barriers were detected for the living compared to the print format, *p* < 0.001. In contrast, external and attitude-related barriers exhibited higher agreement levels for the print compared to the living format of the schizophrenia guideline (*p* < 0.001). For complete test statistics, see Table 2.

*Group comparisons—age:* Kruskal–Wallis tests showed significant differences between age groups concerning attitude-related barriers towards the concept of a living guideline. Younger healthcare professionals (20–34 years) perceived less attitude-related barriers to the living guideline than older healthcare professionals (50–66 years), *p* = 0.019. No significant differences were found regarding knowledge-related and external barriers between age groups (*p* ≥ 0.070). For complete test statistics, see Table 3 and Supplementary Table 5 for post hoc tests.

**Table 3:** Comparisons between age groups: Barriers and facilitating factors in guideline utilization for the print and living format of the guideline for schizophrenia

										KWT		
	Young (20–34 years)			Middle-aged (35–49 years)			Older (50–66 years)					
	*N*	Mdn	M (SD)	*N*	Mdn	M (SD)	*N*	Mdn	M (SD)	H	df	*p*
**Barriers**												
*Knowledge-related* [Q42, Q43, Q45, Q52, Q46]												
Print	122	2.20	2.39 (0.72)	103	2.20	2.42 (0.79)	95	2.20	2.37 (0.78)	0.22	2	0.898
Living	123	3.40	3.37 (0.47)	104	3.45	3.50 (0.52)	99	3.20	3.34 (0.62)	5.33	2	0.070
*Attitude-related* [Q44, Q47, Q48, Q49]												
Print	123	2.25	2.17 (0.59)	103	2.25	2.25 (0.59)	97	2.25	2.18 (0.54)	1.13	2	0.569
Living	123	2.00	1.95 (0.63)	104	2.00	2.12 (0.64)	97	2.00	2.18 (0.55)	8.21	2	0.016
*External* [Q50, Q51, Q53, Q54, Q55]												
Print	122	3.00	3.08 (0.48)	103	3.00	3.04 (0.46)	95	3.00	3.07 (0.49)	0.38	2	0.827
Living	122	3.00	2.86 (0.51)	103	2.80	2.89 (0.43)	95	2.80	2.89 (0.44)	0.11	2	0.944
**Facilitating factors**												
*Knowledge-related* [Q56, Q61, Q68]												
Print	119	4.00	3.95 (0.58)	102	3.83	3.83 (0.60)	93	3.67	3.78 (0.54)	5.45	2	0.066
Living	119	4.00	4.08 (0.59)	102	4.00	4.00 (0.63)	93	4.00	3.91 (0.57)	4.89	2	0.087
*Attitude-related* [Q60, Q62, Q69]												
Print	118	3.67	3.81 (0.58)	99	3.67	3.64 (0.60)	93	3.67	3.61 (0.49)	8.34	2	0.015
Living	118	4.00	3.91 (0.57)	99	3.67	3.67 (0.60)	93	3.67	3.63 (0.51)	14.75	2	0.001
*External* [Q57, Q58, Q59, Q63, Q64, Q65, Q66, Q67, Q70]												
Print	119	3.89	3.83 (0.46)	102	3.67	3.65 (0.52)	93	3.56	3.55 (0.47)	20.20	2	< 0.001
Living	119	3.89	3.90 (0.42)	102	3.78	3.75 (0.53)	93	3.67	3.62 (0.49)	20.43	2	< 0.001

*Note. Print* = guideline for schizophrenia in the print-/pdf-format; *Living* = living guideline for schizophrenia. As no German living guideline for mental disorders existed at the time of the study, participants were asked to answer the questions based on the visual and written description / presentation of the concept of living guidelines. Agreement was assessed by a 5-point Likert scale (1 = *strongly disagree* to 5 = *strongly agree*). *N* = number of participants, *M* = means, *SD* = standard deviations*, Mdn* = medians, *KWT* = Kruskal–Wallis test, *H* = H-value, *df* = degrees of freedom. Number of question is displayed in square brackets. The complete questionnaire appears in the supplement. For subgroup analyses see Supplementary Table 5

*Group comparisons—profession:* Kruskal–Wallis tests indicated significant differences among professions regarding knowledge-related barriers of the print version (see Table 4). Psychosocial therapists and caregivers were more influenced by knowledge-related barriers than medical doctors and psychologists/psychotherapists regarding the print version (*p*s < 0.001). In terms of attitude-related barriers (print format), psychosocial therapists exhibited higher confirmation rates than psychologists/psychotherapists (*p* = 0.002) and medical doctors (*p* = 0.002). No significant differences among professions were found for external barriers of the print version. Concerning the living guideline, psychosocial therapists and caregivers reported more attitude-related barriers than medical doctors and psychologists/psychotherapists (*p* ≤ 0.004). Additionally, caregivers appeared to be more constrained by external barriers than medical doctors (*p* = 0.001).

**Table 4:** Mean response comparisons between professions regarding the barriers and facilitating factors in guideline utilization for both print and living format of the guideline for schizophrenia

																Kruskal–Wallis test		
	Total			PSY			MED			PST			CG					
	*N*	Mdn	M (SD)	*N*	Mdn	M (SD)	*N*	Mdn	M (SD)	*N*	Mdn	M (SD)	*N*	Mdn	M (SD)	H	df	*p*
**Barriers**																		
*Knowledge-related* [Q42, Q43, Q45, Q52, Q46]																		
Print	329	2.40	2.41 (0.75)	63	2.20	2.31 (0.69)	163	2.00	2.10 (0.61)	39	3.20	3.14 (0.60)	54	3.00	2.94 (0.70)	87.43	3	< 0.001
Living	329	3.40	2.41 (0.75)	63	3.40	3.46 (0.49)	163	3.40	3.36 (0.54)	39	3.50	3.55 (0.51)	54	3.60	3.43 (0.60)	3.86	3	0.277
*Attitude-related* [Q44, Q47, Q48, Q49]																		
Print	327	2.25	2.21 (0.59)	63	2.00	2.14 (0.49)	163	2.00	2.11 (0.55)	38	2.50	2.53 (0.63)	53	2.25	2.37 (0.53)	19.18	3	< 0.001
Living	327	2.00	2.08 (0.62)	63	2.00	1.92 (0.55)	163	2.00	1.96 (0.58)	38	2.25	2.36 (0.61)	53	2.25	2.38 (0.57)	31.78	3	< 0.001
*External* [Q50, Q51, Q53, Q54, Q55]																		
Print	323	3.00	3.07 (0.47)	63	3.00	3.06 (0.54)	162	3.00	3.06 (0.48)	36	3.00	3.12 (0.40)	52	3.00	3.09 (0.40)	0.27	3	0.965
Living	323	2.80	2.88 (0.46)	63	2.80	2.87 (0.44)	162	2.80	2.81 (0.48)	36	3.00	2.91 (0.42)	52	3.00	3.08 (0.41)	13.50	3	0.004
**Facilitating factors**																		
*Knowledge-related* [Q56, Q61, Q68]																		
Print	316	4.00	3.86 (0.58)	62	3.67	3.82 (0.58)	161	4.00	3.88 (0.55)	35	4.00	3.92 (0.61)	49	4.00	3.86 (0.62)	1.73	3	0.631
Living	316	4.00	4.00 (0.60)	62	4.00	4.00 (0.58)	161	4.00	4.06 (0.57)	35	4.00	3.97 (0.62)	49	4.00	3.94 (0.63)	2.66	3	0.447
*Attitude-related* [Q60, Q62, Q69]																		
Print	312	3.67	3.69 (0.56)	61	3.67	3.67 (0.58)	160	3.67	3.70 (0.56)	35	3.67	3.69 (0.58)	48	3.67	3.74 (0.50)	0.47	3	0.926
Living	312	3.67	3.75 (0.57)	61	3.67	3.75 (0.60)	160	3.67	3.77 (0.58)	35	3.67	3.71 (0.57)	48	3.67	3.75 (0.51)	0.51	3	0.917
*External* [Q57, Q58, Q59, Q63, Q64, Q65, Q66, Q67, Q70]																		
Print	316	3.72	3.69 (0.50)	62	3.67	3.67 (0.52)	161	3.67	3.65 (0.52)	35	3.78	3.74 (0.44)	49	3.89	3.79 (0.39)	4.27	3	0.234
Living	316	3.78	3.77 (0.49)	62	3.78	3.75 (0.50)	161	3.78	3.75 (0.51)	35	3.78	3.77 (0.45)	49	3.89	3.84 (0.43)	1.383	3	0.709

*Note. Print* = guideline for schizophrenia in the print-/pdf-format; *Living* = living guideline for schizophrenia. As no German living guideline for mental disorders existed at the time of the study, participants were asked to answer the questions based on the visual and written description / presentation of the concept of living guidelines. Agreement was assessed by a 5-point Likert scale (1 = strongly disagree to 5 = strongly agree). *N* = number of participants, *M* = means, *SD* = standard deviations, *Mdn* = medians, *KWT* = Kruskal–Wallis test, *H* = H-value, *df* = degrees of freedom. Number of question is displayed in square brackets. Total = all participants included, PSY = psychologists/psychotherapists, MED = medical doctors, PST = psychosocial therapists, CG = caregivers. The complete questionnaire appears in the supplement. For subgroup analyses see Supplementary Table 6

No significant differences among professions were found for knowledge-related barriers of a living guideline as well as external barriers of the print version of the schizophrenia guideline. For complete test statistics, see Table 4 and Supplementary Table 6.

### Facilitating factors in guideline implementation

The explored facilitating factors were analogue to the barriers assigned to Cabana’s knowledge–attitude–behavior framework. See Table 5 for an overview.

**Table 5:** Facilitating factors in guideline utilization for the print and living format of the guideline for schizophrenia

		% Yes	Mdn	M	SD	Wilcoxon test	
						Z	*p*
**Knowledge-related facilitating factors**							
Firm implementation of guidelines in the curriculum [Q61] (*n* = 310)	*Print*	82.9%	4.00	4.12	0.73	1.93	0.054
	*Living*	84.5%	4.00	4.15	0.71		
Notifications in case of updates [Q68] (*n* = 309)	*Print*	83.2%	4.00	4.15	0.77	3.07	0.002
	*Living*	85.1%	4.00	4.21	0.75		
Trainings for professionals [Q56] (*n* = 314)	*Print*	51.3%	4.00	3.34	1.05	7.51	< 0.001
	*Living*	69.7%	4.00	3.69	0.99		
Mean—knowledge-related facilitators* (*n* = 309)	*Print*	72.5%	4.00	3.87	0.56	– 7.32	< 0.001
	*Living*	79.8%	4.00	4.02	0.58		
**Attitude-related facilitating factors**							
Increased consideration of clinical conditions [Q62] (*n* = 310)	*Print*	74.2%	4.00	3.98	0.77	2.76	0.006
	*Living*	77.7%	4.00	4.05	0.75		
Involvement of clinicians in guideline Development [Q60] (*n* = 310)	*Print*	50.3%	4.00	3.52	0.81	1.95	0.051
	*Living*	52.9%	4.00	3.56	0.80		
Promotion of guideline benefits (e.g., advertisement) [Q69] (*n* = 309)	*Print*	55.3%	4.00	3.58	0.92	3.00	0.003
	*Living*	58.6%	4.00	3.63	0.91		
Mean—attitude-related facilitators* (*n* = 309)	*Print*	63.1%	3.67	3.69	0.56	– 3.81	< 0.001
	*Living*	59.9%	3.67	3.75	0.57		
**External facilitating factors**							
Feedback from patients (e.g., on drug tolerability) [Q57] (*n* = 314)	Print	71.7%	4.00	3.85	0.82	3.32	< 0.001
	Living	76.4%	4.00	3.93	0.77		
Trainings for patients and relatives [Q58] (*n* = 314)	Print	61.1%	4.00	3.69	0.88	31.63	0.018
	Living	65.0%	4.00	3.73	0.87		
Possiblity to use the guideline for shared-decision-making [Q59] (*n* = 310)	Print	60.3%	4.00	3.66	0.85	5.48	< 0.001
	Living	70.3%	4.00	3.86	0.79		
Quality management [Q63] (*n* = 309)	Print	31.0%	3.00	3.17	0.87	2.77	0.006
	Living	33.5%	3.00	3.22	0.90		
Provision of electronic devices (tablets, smartphones) [Q64] (*n* = 309)	Print	61.6%	4.00	3.72	1.05	6.23	< 0.001
	Living	73.9%	4.00	3.98	0.94		
Simpler language [Q65] (*n* = 309)	Print	39.2%	3.00	3.18	1.02	– 1.48	0.139
	Living	36.6%	3.00	3.15	1.00		
Short and concise versions with essential treatment recommendations [Q66] (*n* = 309)	Print	69.9%	4.00	3.87	1.00	0.87	0.387
	Living	71.8%	4.00	3.90	0.99		
Treatment checklists [Q67] (*n* = 309)	Print	88.3%	4.00	4.30	0.75	1.19	0.234
	Living	89.6%	4.00	4.32	0.72		
Tailored guideline versions (profession, specification) [Q70] (*n* = 309)	Print	64.4%	4.00	3.76	0.97	3.22	0.001
	Living	67.3%	4.00	3.81	0.96		
Mean—external facilitators* (*n* = 309)	Print	60.8%	3.78	3.69	0.49	– 6.31	< 0.001
	Living	64.9%	3.78	3.77	0.49		

*Note. Print* = guideline for schizophrenia in the print-/pdf-format; *Living* = living guideline for schizophrenia*.* Agreement on barriers was assessed by a 5-point Likert scale (1 = *strongly disagree* to 5 = *strongly agree*). *% Yes* represents the percentage of participants, who *agreed* or *strongly agreed* to the statement. *N*  number of participants, *M*  means, *SD*  standard deviations, *Mdn*  medians, *Z*  standard score. As no German living guideline for mental disorders existed at the time of the study, participants were asked to answer the questions based on the visual and written description / presentation of the concept of living guidelines. Numbers of questions are displayed in square brackets. The complete questionnaire is shown in the supplement. *The variables represent the mean agreement rate on items of the corresponding categories “Knowledge-Attitude-Behavior Framework”

The surveyed mental healthcare professionals considered the provision of treatment checklists (living: 90%; print: 88%) as the main facilitating factor in the implementation of the schizophrenia guideline for both formats (living and print), followed by notifications in case of updates (living: 85%; print: 83%) and a firm implementation of the specific guideline in the curriculum (living: 85%; print: 83%), see Table 5.

*Group comparisons—print versus living:* Wilcoxon tests for dependent samples indicate a greater need for knowledge-related and external facilitating factors among mental healthcare professionals in the implementation of the living guideline compared to the print format *(p* < 0.001), see Table 5. In terms of the print version, there was a higher reported need for attitude-related facilitators (*p* < 0.001).

*Group comparisons—age:* Younger healthcare professionals reported a higher need for attitude-related facilitating factors than older (print: *p* = 0.024; living: *p* = 0.001) and middle-aged healthcare professionals (living: *p* = 0.010). Regarding external facilitating factors, younger professionals expressed a higher confirmation rate than older (print: *p* ≤ 0.001; living:* p* ≤ 0.001) and middle-aged professionals (print: *p* = 0.008). For complete test statistics, see Table 3 and Supplementary Table 5 for post hoc tests.

*Group comparisons—profession:* Kruskal–Wallis tests found no significant results between professions concerning facilitating factors for the print and living guideline format. Overall, results indicated an agreement (all *M*s > 3) of all professions requiring more knowledge-related, attitude-related and external facilitating factors in guideline utilization for both formats print and living (see Table 4).

### Preferences in the use of living guidelines

Concerning preferences in using an upcoming living guideline, 97% of the participants would prefer an update at least annually of the recommendations in the living guideline (see Supplementary Table 8). Moreover, about 38% of the respondents would like to be notified immediately of new and relevant research findings, whereas only 3% do not want to receive notifications. Less than 10% of the participants reported that an annual update of recommendations or references to new research findings would evoke pressure to constantly adjusting treatment. In contrast, about 74% of the participants considered this update as a relief, because there would be more confidence that the current treatment of patients is according to the ‘state of the art’. Approximately 17% of the respondents reported to use other formats than guidelines (e.g., textbooks) to learn about evidence-based treatment (agreed and strongly agreed), whereas about 33% did not prefer other formats to guidelines (disagreed and strongly disagreed). In order to learn about appropriate treatment options about 15% stated to use guidelines, scientific journals or exchange with colleagues, while 34% answered to use professional literature. Most of the surveyed healthcare professionals (63%) stated to at least occasionally use digital tools/apps, whereas only 16% reported never having used digital tools in everyday clinical practice. For an overview of the descriptive characteristics, see Supplementary Table 7. An overview of our presented results regarding barriers, facilitators as well as preferences and differences between professions and age groups is shown in Table 6.

**Table 6:** Overview of our results regarding barriers, facilitators as well as preferences and differences between professions and age groups

	Print format	Living guideline
**Knowledge-related barriers**		More knowledge-related barriers compared to the print format
Professions	CG, PST more influenced than MD, PSY	
Age groups	No significant differences between age-groups for both formats	
**Attitude-related barriers**	More attitude-related barriers	
Professions	CG, PST more influenced than MD, PSY for both formats	
Age groups		Younger (20–34 years) experience less attitude-related barriers than older (50–66 years) health-care professionals
**External barriers**	More external barriers	
Professions		CG more influenced than MD
Age groups	No significant differences between age-groups for both formats	
**Knowledge-related facilitators**		More knowledge-related facilitators needed compared to the print format
Professions	Agreement of all professions requiring more knowledge-related facilitators for both formats	
Age groups	No significant differences between age-groups for both formats	
**Attitude-related facilitators**	More attitude-related facilitators needed compared to the living format	
Professions	Agreement of all professions requiring more attitude-related facilitators for both formats	
Age groups	Younger need more facilitators than older professionals for both formats	
**External facilitators**		More external facilitators needed compared to the print format
Professions	Agreement of all professions requiring more external facilitators for both formats	
Age groups	Younger (20–34 years) need more facilitators than older (50–66 years) professionals for both formats	
*Preferences (living guideline)*	Annual update, immediate notifications in case of updates, relief not to oversee what is state of the art	

Note *PSY*  psychologists/psychotherapists, *MD*  medical doctors, *PST*  psychosocial therapists, *CG*  caregivers

## Discussion

This study displays barriers and facilitators in guideline implementation for both: the current schizophrenia guideline in the print and the concept of a living format. To our knowledge, this is the first study drawing attention to obstacles and facilitators in implementing a living guideline.

The most frequently mentioned barrier regarding the print version was lack of time resources, followed by insufficient training in guidelines use and too long or complex versions. Regarding the living guideline, the most frequently cited barriers were knowledge-related, which could be explained by the new format and the fact that no living guideline for mental disorder is available yet [20]. In contrast, as the print version was found to be more vulnerable to attitude-related and external barriers, one possible solution to overcome this situation could be the development of living guidelines. This notion that living guidelines could be a worthwhile tool to improve guideline adherence is supported by the often reported facilitating factors such as notifications in case of updates, more guideline trainings, treatment checklists and shorter versions as these factors can be more easily addressed with a living guideline usually embedded in a flexible digital system such as MAGICapp [15]. Moreover, lack of time is one of the most frequently reported barriers to guideline adherence in general [21–24] and regarding the print version in our survey. Living guidelines may resolve this barrier making digitalized learning easier (e.g., by directly linking guidelines to other sources of evidence) and saving time concurrently. This consideration is underlined by a recent study about dissemination of psychiatric practice guidelines, which found web-based courses about guideline knowledge more satisfying and as effective as face-to-face courses [25].

When examining possible age differences, younger professionals reported significantly less attitude-related obstacles than older professionals in the context of a living guideline. This may be due to the circumstance that participants of younger age may be more experienced and thus more confident in using technical devices and apps in their everyday life [26]. In respect to facilitating factors, younger participants expressed a higher need to attitude-related and external facilitators than older and middle-aged participants for both formats. One explanation could be that younger people will be more affected in their further professional lives by the increasing prevalence of living guidelines [12, 27]. Consequently, they might have a greater interest in possible solutions for guideline implementation, also as younger professionals are more inclined to use guidelines [22, 28].

Several studies show profession-specific differences in guideline implementation [6, 22, 27]. Therefore, a closer look at profession-specific obstacles and facilitators to guideline adherence is essential. Regarding the print version of the schizophrenia guideline, there was a consensus among professions with respect to external barriers. However, caregivers and psychosocial therapists stated to be more influenced by knowledge- and attitude-related barriers than medical doctors and psychologists, which could explain the lower implementation rate of the schizophrenia guideline in these professions [6]. In detail, about 67% of psychosocial therapists and 35% of caregivers stated having a lack of experience with the print version of the guideline, while this was the case for only 8% of physicians. Moreover, 13% of psychosocial therapists and only 6% of medical doctors reported a lack of benefit for their clinical work (see Supplementary Table 6). The German schizophrenia guideline has relatively more recommendations concerning the everyday clinical work of medical doctors than of psychosocial therapists or caregivers [7], thus, the idea could prevail that the recommendations might be less relevant for the respective professional groups in everyday clinical practice. This explanation could also account for the concept of a living guideline where psychosocial therapists reported more attitude-related and caregivers additionally more external obstacles to guideline adherence.

The depicted profession-specific barriers to guideline adherence for both formats accentuate the need for target-specific implementation strategies [6, 22, 29–32]. In general, evidence regarding effective implementation strategies is heterogeneous and insufficient [2, 33, 34]. However, there is agreement that the passive introduction of guidelines alone does not improve implementation [5, 22]. Rather, a structured implementation is required considering the barriers and facilitators across all stages of behavior change [5, 6].

Our results show a high agreement for the need of facilitating factors among mental healthcare professionals for both formats. Knowledge-related facilitators such as notifications in case of updates may be well encountered with web-based living guidelines. This corresponds to our findings that most of the surveyed participants wished to be updated immediately in case of new research findings and would be relieved as they could be sure not to overlook what is state of the art (76%). Regarding attitude-related facilitators, healthcare professionals regarded an increased consideration of clinical conditions with multimorbid patients as helpful, while guidelines often do not consider this comprehensively [22, 24]. This could be improved with the concept of living guidelines in case they are incorporated in a web-based environment (e.g., MAGICapp) as they can be directly linked to other specific guidelines. Web-based tools can further provide descriptive illustrations for shared decision-making as well as shorter and more profession-specific, tailored versions (external facilitators).

Overall, our results show that many of the expressed helpful strategies to guideline implementation can be addressed more easily with the concept of living guidelines than with classic print versions. As more than half of the surveyed healthcare professionals (63%) already apply digital tools/apps in their everyday clinical life, living guidelines seem to be a promising tool to improve guideline adherence.

There are some limitations concerning the results of our study. First, we cannot exclude that participants took part in the study several times as we did not apply tracking of IT addresses. This would not have been compatible with the given regulations on data protection. However, participants were explicitly asked to answer the questionnaire only once. Second, as a living guideline for schizophrenia is not available yet, the participants’ answers were based on presented screenshots of the schizophrenia guideline in the online environment of MAGICapp. This can possibly lead to bias, as it is difficult to represent the holistic concept of a living guideline with screenshots. Moreover, the depicted screenshots were taken from the evidence ecosystem MAGICapp. However, other digital tools for living guidelines exist and could result in a different evaluation. Third, we detected significant differences between professions (age, gender, work setting and working experience) and between included and excluded participants (gender, profession, setting, age) regarding demographic information (see Supplementary Tables 2, 3, 4). As a large proportion of the excluded participants did not indicate which professional group they belonged to, *p* < 0.001 (“Other”, see Supplementary Table 2), there is probably a significant effect on the proportion of included compared to excluded medical doctors, *p* < 0.001. Moreover, the drop-out group was significantly younger than the included group. However, the differences between included participants and drop-outs were subtle without a clear pattern of a systematic bias. However, about one-third of the participants started the survey but did not complete it, possibly resulting in a bias regarding the results and could be explained by a lack of time to answer the comprehensive survey.

## Conclusion

Various barriers exist for both guideline formats and a high need for facilitators was expressed across all professions. Many of the mentioned obstacles and facilitators may be more easily addressed with living guidelines embedded in online environments such as the evidence ecosystem MAGICapp. However, living guidelines themselves are fraught with many predominantly knowledge-related barriers. Thus, the introduction of these new formats alone cannot lead to sustainable behavior change regarding guideline adherence, in fact all stages of behavior change must be considered, including the identification of knowledge-, attitude- and behavior-related barriers as well as facilitating factors.

As living guidelines are becoming increasingly widespread in medicine [12, 27], our findings represent first insights into barriers, facilitators and preferences which can enhance a successful implementation of a (living) guideline.

### Supplementary Information

Below is the link to the electronic supplementary material.

Supplementary file1 (DOCX 637 KB)

## Data Availability

The datasets used and/or analysed during the current study are available from the corresponding author on reasonable request.
